# Search for Reliable Circulating Biomarkers to Predict Carotid Plaque Vulnerability

**DOI:** 10.3390/ijms21218236

**Published:** 2020-11-03

**Authors:** Núria Puig, Elena Jiménez-Xarrié, Pol Camps-Renom, Sonia Benitez

**Affiliations:** 1 Department of Biochemistry and Molecular Biology, Faculty of Medicine, Building M, Autonomous University of Barcelona (UAB), 08193 Cerdanyola del Vallès, Barcelona, Spain; npuigg@santpau.cat; 2 Cardiovascular Biochemistry, Biomedical Research Institute Sant Pau (IIB-Sant Pau), 08041 Barcelona, Spain; 3 Stroke Unit, Department of Neurology, Hospital de la Santa Creu i Sant Pau (IIB-Sant Pau), 08041 Barcelona, Spain

**Keywords:** carotid atherosclerosis, ischemic stroke, atherothrombotic stroke, biomarkers, lipoproteins, lipids, inflammation, plaque vulnerability

## Abstract

Atherosclerosis is responsible for 20% of ischemic strokes, and the plaques from the internal carotid artery the most frequently involved. Lipoproteins play a key role in carotid atherosclerosis since lipid accumulation contributes to plaque progression and chronic inflammation, both factors leading to plaque vulnerability. Carotid revascularization to prevent future vascular events is reasonable in some patients with high-grade carotid stenosis. However, the degree of stenosis alone is not sufficient to decide upon the best clinical management in some situations. In this context, it is essential to further characterize plaque vulnerability, according to specific characteristics (lipid-rich core, fibrous cap thinning, intraplaque hemorrhage). Although these features can be partly detected by imaging techniques, identifying carotid plaque vulnerability is still challenging. Therefore, the study of circulating biomarkers could provide adjunctive criteria to predict the risk of atherothrombotic stroke. In this regard, several molecules have been found altered, but reliable biomarkers have not been clearly established yet. The current review discusses the concept of vulnerable carotid plaque, and collects existing information about putative circulating biomarkers, being particularly focused on lipid-related and inflammatory molecules.

## 1. Introduction

Stroke is a leading cause of death, disability, and dementia worldwide. It represents the third cause of mortality in western countries. In addition, more than 20% of patients suffer a stroke recurrence within 5 years of follow-up, increasing the risk of severe disability [1]. Eighty percent of the total of strokes are ischemic [2], and approximately 20% of them are caused by large-artery or large-vessel atherosclerosis [3], with plaques in the internal carotid artery (ICA) being the most frequently involved ones [4]. Ischemic strokes associated with atherosclerosis are globally named atherothrombotic strokes [5]. There are two main mechanisms related to the pathogenesis of atherothrombotic stroke: plaque rupture, which leads to thrombus formation and subsequent distal embolism [6]; and hemodynamic insufficiency, which may be attributed to progressive vessel occlusion caused by atherosclerotic plaque progression [7].

Despite the recent advances in medical and surgical treatments, the cerebrovascular burden of atherosclerosis remains considerably high [8]. The management of patients with a recent stroke and carotid atherosclerosis depends to a large extent on the degree of stenosis, as determined by carotid imaging techniques. However, the degree of stenosis alone is not a sufficient basis when deciding on the best treatment in some common clinical situations, such as in women with moderate carotid stenosis (50–69%), patients presenting a monocular vision loss [9,10], or in cases with suspected vulnerable plaques causing less than 50% of stenosis [11], among others. Thus, there is a need for complementary biomarkers that may help in classifying high-risk patients and plaque vulnerability.

Before a symptomatic ischemic stroke occurs, an atherosclerotic lesion frequently suffers from asymptomatic microruptures that lead to microemboli [12]. As a consequence, inflammation and lipid-related molecules may diffuse from the lesion milieu and are released into the systemic circulation [13]. This phenomenon brings us the opportunity to determine which molecules are the major predictors of atherosclerotic burden, ischemic stroke, plaque vulnerability, and disease progression. The discovery of reliable plasma biomarkers may, in the future, complement the data obtained through carotid imaging.

This review discusses the concept of vulnerable carotid plaque, and it presents the existing information on the putative circulating biomarkers for carotid atherosclerosis, with a focus on lipid-related and inflammatory molecules. This review also discusses the relationship between these biomarkers and the occurrence/recurrence of symptoms, as well as the putative use of these biomarkers in the clinical practice.

## 2. Atherosclerosis and Plaque Vulnerability

Atherosclerosis is an inflammatory process triggered by lipoprotein retention in the artery wall. The overlying deposition of lipids leads to narrowing (stenosis) of arteries [14]. Atherosclerotic plaque formation is a slow, progressive process that may begin in childhood and remain silent over many years. It mainly occurs in large- and medium-sized arteries, particularly in zones of curvatures or bifurcation with turbulent flow (abdominal aorta, coronary, and carotid artery). Particularly, low shear stress zones, with non-laminar flow, are more prone to plaque development and rupture. Atherosclerosis is a multifactorial disease, wherein several risk factors contribute to its development, mainly advanced age, male gender, hypertension, smoking, diabetes, hypercholesterolemia, infection, autoimmunity, a diet high in fat and cholesterol, sedentary lifestyle, family history, genetic disposition and mental/social stress [15].

Given that atherosclerotic lesions exhibit a long-term development, they can be found in several stages, from an initial, asymptomatic, and non-stenotic stage to an advanced phase during which thrombi are formed, leading to embolism [16]. In this context, the main stages are as follows:

Fatty streak: The earliest sign of atherosclerosis is endothelial dysfunction which favors low-density lipoprotein (LDL) entry into the subendothelial space. In this microenvironment, the generation of modified LDL triggers the recruitment of monocytes, their differentiation into macrophages, the formation of lipid-loaded foam cells, and the induction of an inflammatory response.

Fibroproliferative lesion: Further inflammation allows the infiltration of more leukocytes, leading to plaque development. The fibrous cap is formed by the activation of smooth muscle cells that are recruited to the intima and that which secrete connective tissue.

Advanced lesion: Inflammation is enhanced triggering cell apoptosis and death, and the formation of a lipid-rich necrotic core. Additionally, degradation of the extracellular matrix occurs.

More advanced lesion: This condition is characterized by increased cell necrosis, severe inflammation, calcification, cholesterol crystals, neo-vascularization, and thinning of fibrous cap, leading to plaque rupture in the shoulder regions. In the latest stage of the process, a thrombus generation may be triggered by the exposure of the lipid core or by molecules released by cells.

Therefore, in the earliest stage of atherosclerosis, LDL is retained within the subendothelial space, where it is modified by oxidative stress and enzymatic activities, and it acquires inflammatory properties. In endothelial cells, modified LDL induces the expression of adhesion molecules and chemoattractant cytokines (chemokines), both involved in the recruitment of circulating leukocytes to the lesion area. Infiltrated monocytes are then differentiated into macrophages, which, in response to modified LDL, elicit an enhanced inflammatory response by releasing more chemokines, other cytokines, such as tumor necrosis factor alpha (TNF-α) and interleukin-1β (IL-1β), and growth factors. This response promotes the recruitment and activation of SMCs, which secrete connective tissue and thus contribute to the generation of the fibrous cap. SMCs and mainly macrophages are able to uptake modified LDL becoming lipid-loaded foam cells. At this stage, when the counteracting response against inflammation is defective or insufficient, atherosclerotic plaques that had remained stable for a long time may progress and become unstable, as reviewed by Tabas et al. [17].

A stable plaque shows a thick fibrous cap versus lipid core and exhibits a generally calcified state [18]. By contrast, an unstable plaque is characterized by necrosis, thin fibrous cap covering a lipid-necrotic core, infiltration of inflammatory cells, and presence of weak microvessels. In a situation of defective inflammation resolution, a defective clearance of dead cells is triggered, leading to the generation of the necrotic core. In its turn, necrotic macrophages release matrix metalloproteinases (MMPs) [19] and other proteolytic enzymes that hydrolize extracellular matrix and cause loss of thickness of the fibrous cap. In addition, neovessels are formed due to plaque thickening and the presence of angiogenic factors. They are weak and permeable and thus allow an increased blood cell infiltration, leading to intraplaque hemorrhage [20]. In this advanced stage of an unstable plaque, necrotic macrophages also release lipids, inflammatory and pro-thrombotic molecules, leading eventually to plaque vulnerability and thrombosis, and, in the case of carotid artery atherosclerosis, to a cerebrovascular event.

**Figure 1:**
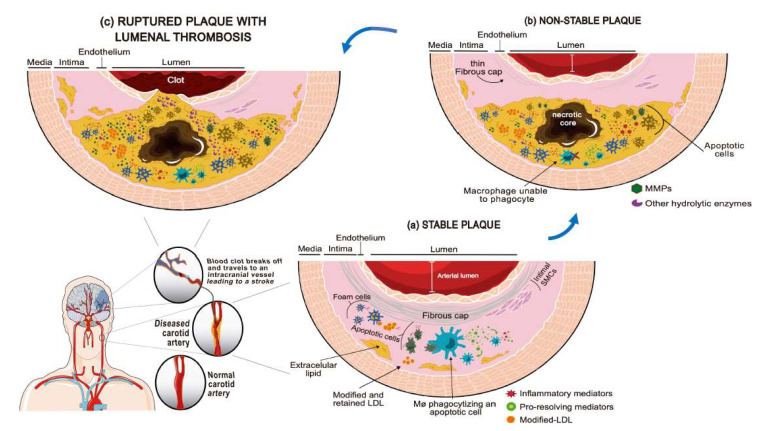
Progression of carotid plaque leading to ischemic stroke. Atherosclerosis in the carotid artery is the main cause of atherothrombotic ischemic stroke. Atherosclerosis may develop for years without symptoms for as long as the plaque remains stable. (**a**) When the mechanisms counteracting inflammation are overwhelmed, the balance to inflammatory processes is favored, leading to necrosis and release of inflammatory mediators and proteolytic enzymes that degrade the fibrous cap. The plaque then becomes unstable. (**b**) The unstable atherosclerotic plaques may break and then coagulation is triggered, eventually leading to thrombosis and brain ischemic events (**c**).

Figure 1 illustrates the progression from stable to unstable carotid plaque leading to ischemic stroke, and also shows the main molecular and cell players, including modified LDL and macrophages.

## 3. Carotid Plaque Vulnerability and Ischemic Stroke

The most frequent mechanism involved in the pathogenesis of atherothrombotic stroke is the rupture of a carotid plaque with the subsequent thrombus formation and distal embolism (Figure 1). The treatment for atherothrombotic stroke in its acute phase is similar to that for the other stroke subtypes. However, the secondary prevention after an atherosclerosis-related stroke varies and involves two different approaches that are often complementary: medical treatment and revascularization therapies, which are based on carotid stenting or on surgical carotid endarterectomy (CEA). The benefits of CEA in symptomatic patients with high-grade carotid stenosis were proven by the large-scale randomized carotid surgical trials conducted in the 1990s (NASCET Trial 1991 and ESCT Trial 1998). Although these trials have reported positive results, some open questions remain unsolved up to these days. For instance, the suitable treatment for women with moderate stenosis, for patients not revascularized within the first 15 days, or for patients presenting two potential stroke etiologies at the same time, remains unknown. All these clinical situations are relatively frequently encountered in the current practice, and use of the degree of stenosis alone as a basis when deciding on the best management is insufficient [10].

Symptomatic stroke patients with carotid arteriosclerosis usually have unstable rupture-prone lesions presenting specific morphological and histological features [21]. These features can be studied by imaging techniques, including ultrasonography, computerized tomography, or magnetic resonance [22,23,24]. Doppler ultrasound provides some interesting information related to plaque vulnerability, including plaque echolucency that is associated with increased lipid and inflammatory molecule content or microemboli detection that is related to the risk of stroke recurrence [12], among others. For example, contrast-enhanced ultrasound (CEUS) allows for the detection of intraplaque neovascularization and it has been related to the risk of stroke recurrence independently of the degree of stenosis [23]. Furthermore, there has been a significant increase in the use of three-dimensional carotid ultrasound reconstruction software for estimating carotid plaque burden, which allows a fast and reproducible volumetric study of the plaque, and a better risk stratification of individuals [25]. Computed tomography angiography detects the presence of ulcerated plaque with higher sensitivity than ultrasonography [26]. Meanwhile, high-resolution magnetic resonance allows the detection of vulnerability features such as lipid-rich core necrosis, fibrous cap thinning/rupture, and intraplaque hemorrhage [24]. In the field of carotid imaging, positron emission tomography-computed tomography with 18F-fluorodeoxyglucose (18-FDG PET) deserves a special mention. 18-FDG PET can estimate plaque inflammation in vivo based on the radiotracer uptake by active intraplaque macrophages [27]. Therefore, PET provides important pathophysiological information regarding a plaque beyond the degree of stenosis, along with information on the other morphological features. Carotid plaque inflammation detected by 18-FDG PET was found to be higher in symptomatic than in asymptomatic patients [28], and it could predict recurrence of ischemic stroke, regardless of the degree of stenosis [29]. Eventually, it was described that a risk score that combines stenosis degree and 18-FDG PET values improves the identification of early recurrent stroke [30].

In spite of the ultimate advances in carotid imaging, predicting plaque vulnerability remains challenging. In this regard, plasma biomarkers could provide adjunctive criteria, allowing for a better diagnosis, risk stratification, and clinical management.

## 4. Search for Circulating Biomarkers in Atherothrombotic Ischemic Stroke

One of the priorities in the field of ischemic stroke prevention is finding new biomarkers that may help in assessing the vulnerability of atherosclerotic plaques. The identification of biomarkers will help in answering the two key questions that arise whenever we find an atherosclerotic plaque in the carotid artery or in the intracranial circulation in the setting of an ischemic stroke: (1) Does a correlation exist between the plaque and the stroke pathogenesis? (2) What is the risk of stroke recurrence? The second question is extremely important in assessing whether a medical treatment alone will be sufficient to prevent new events or whether a revascularization therapy is necessary.

Ideally, putative biomarkers must satisfy several criteria [1], the most important of these are the following: diagnostic specificity and sensitivity, ability to differentiate between stroke subtypes, correlation between biomarker concentrations and a certain outcome, prospective validation, incremental value to that of the existing markers, clinical usefulness, and cost-effectiveness. Several studies have proposed a large number of plasma biomarkers for diagnosis and prognosis of ischemic stroke [31]. However, several of the above-mentioned criteria hinder the establishment of well-defined, robust, and useful biomarkers.

**Figure 2:**
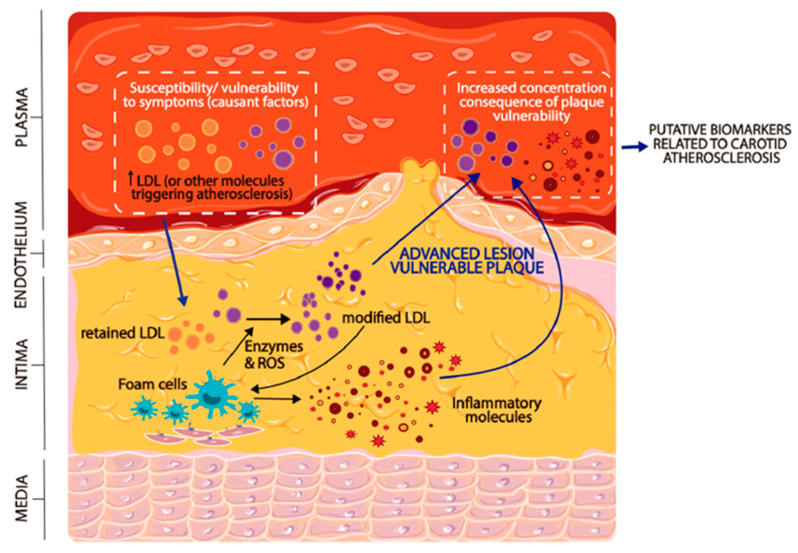
Presence of circulating biomarkers in patients with carotid atherosclerosis. Some molecules in the plasma may be indicative of susceptibility to develop atherosclerosis because of their involvement in the origin and destabilization of atherosclerotic plaque, in carotid as well as in other arteries. LDL and modified LDL may play a key role in this regard by first entering the subendothelial space, where they are presumably further modified, and then promoting foam cell formation and inducing an inflammatory response. Eventually, this phenomenon contributes to plaque vulnerability and to the release into the circulation of inflammatory mediators and highly modified LDL, thereby increasing their plasma concentration, particularly in symptomatic patients. In asymptomatic patients, the concentration of some biomarkers may be higher in high-risk vulnerable patients, because their carotid plaques release part of the lesion-related molecules into the circulation or because they have increased levels of certain molecules (as modified forms of LDL) that are involved in triggering plaque progression.

**Figure 3:**
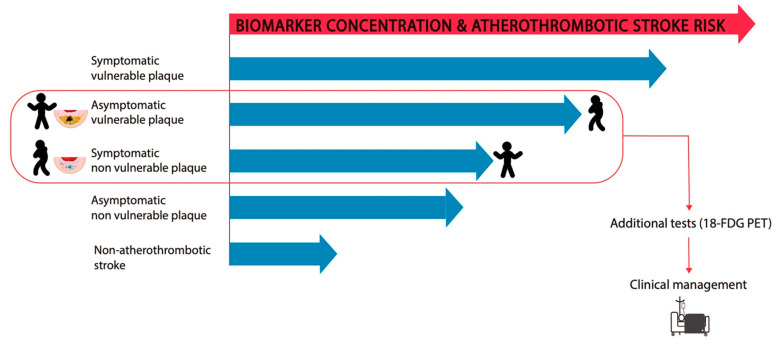
Levels of biomarkers in patients with carotid atherosclerosis and risk of atherothrombotic stroke.

In the specific case of atherothrombotic stroke, the nature of the biomarkers is expected to be inflammatory or lipid-related because of their involvement in atherosclerosis. Figure 2 shows that the plasma concentration of some biomarkers may increase as a consequence of the rupture of a vulnerable plaque, enabling their diffusion from the lesion milieu. In addition, certain biomarkers may be increased in pathological states, and not as a consequence of their release from plaques. In this case, their increased concentration may contribute to high susceptibility to the development of atherosclerotic plaques and/or to the vulnerability and rupture of existing plaques. According to this hypothesis, an increased concentration of plasma biomarkers, regardless of their origin, should be indicative of a high risk for atherothrombotic stroke, as suggested in the hypothetical model in Figure 3. Therefore, patients should be subjected to additional tests to determine the most suitable medical intervention. This knowledge is useful not only in predicting recurrence in symptomatic patients but also in predicting putative future events in asymptomatic patients.

An increased concentration of a biomarker or a set of biomarkers should indicate the occurrence or recurrence of ischemic stroke in asymptomatic and symptomatic patients, respectively. In the absence of atherothrombotic stroke, biomarkers are expected to show the lowest level compared with the levels in other conditions; however, biomarker levels may be higher in asymptomatic/symptomatic patients with stable plaques. When a plaque becomes vulnerable, biomarker concentration is expected to considerably increase, especially in symptomatic patients Evaluation of these biomarkers, together with the assessment of the plaques by neuroimaging, (e.g., 18-FDG PET), will allow for a better stratification of the risk of atherothrombotic stroke. Therefore, these tools will allow the selection of high-risk patients who may become a candidate for CEA.

Currently, individual biomarkers to be targeted routinely remain to be established. In this context, it would be more useful to find alterations in clusters of several biomarkers than to select rigid cutoffs for a single biomarker [32]. On this basis, some approaches have been used to screen for protein/genomic signature and to select putative ischemic stroke- and carotid atherosclerosis-related biomarkers evaluable in clinical practice. Due to easier access to the carotid artery, proteomic studies have been performed not only in the circulation but also within the plaques [33,34], as well as in the secretome of surgically removed plaques [35]. Genomic tools have been used to find gene signatures in peripheral blood mononuclear cells and in whole blood from ischemic stroke patients [36,37]. In carotid plaque, differences have been found not only between symptomatic and asymptomatic patients [38,39] but also between unstable and stable carotid plaque from asymptomatic patients [40].

In summary, in the field of atherothrombotic stroke, the identification in the plasma of reliable and specific biomarkers that are indicative of the carotid plaque vulnerability is essential. This review summarizes the up-to-date data on plasma biomarkers for ischemic stroke, with a focus on carotid atherosclerosis and lipid-related and inflammatory molecules. All the information specifically reported on the atherothrombotic subtype is summarized in Table 1.

## 5. Lipid-related and Lipoproteins Biomarkers

### 5.1. Lipids and Lipoproteins

Several lines of evidence have shown the pivotal role of cholesterol in carotid artery atherosclerosis. Atherosclerotic plaques are lipid-enriched, and their lipid content strongly determines the plaque vulnerability and the rate of stenosis progression. Extracellular cholesterol crystals accumulated in a carotid plaque promote inflammation and erosion of the fibrous cap, eventually leading to plaque vulnerability [113]. Specific composition in lipid species, such as ceramide, contributes to lesion vulnerability [114], since ceramide concentration correlates with inflammatory cytokines, apoptosis, and with histological markers of plaque instability. Lipids accumulated in the carotid lesion may be of intracellular origin or be the consequence of extracellular accumulation of modified lipoproteins, mainly aggregated and fused LDL [59]. The origin of lipoproteins is plasmatic; therefore, high plasma concentrations of cholesterol and LDL are expected to indicate the propensity to develop carotid atherosclerosis, as shown in Figure 2.

Most epidemiological studies have shown a direct correlation between lipid levels and risk of ischemic stroke [32,115], and this association depends on the specific lipid component being considered and on the stroke subtype. In intracerebral hemorrhage (ICH) and in small vessel disease there is increased stroke risk with low cholesterol levels [115]. By contrast, high plasma concentrations of total cholesterol (TC) and LDL-cholesterol (LDLc), are particularly related to atherothrombotic stroke [41,116,117], whereas hypertriglyceridemia is commonly found in patients with ischemic stroke regardless of the etiological subtype. Several lines of evidence have demonstrated the role of LDLc concentration in stroke. For instance, high LDLc concentrations increase the risk of stenosis progression in symptomatic patients with mild to moderate stenosis [43], whereas reduction of TC and LDLc levels by an intensive lipid-lowering therapy reduces the recurrence and future cardiovascular events [118], as well as prevents the progression of carotid atherosclerosis in asymptomatic [119] and symptomatic patients [120]. Statins are the most widely used lipid-lowering treatment and it has been demonstrated that their use in patients with non-cardioembolic strokes (including small-vessel disease and atherothrombotic strokes) reduces the incidence of stroke recurrence and other cardiovascular events. Statins reduce inflammation of the atherosclerotic plaques by lowering the plasmatic levels of LDL, but they also have pleiotropic effects including attenuation of chronic inflammation. In this regard, besides statins, the inhibitor of proprotein convertase subtilisin/kexin 9 (PCSK9), an enzyme involved in LDLc elevation, have demonstrated efficacy in reducing stroke risk. However, the increased serum levels of PCSK9 are associated with carotid atherosclerosis, independently of its LDLc-lowering effect [45,121].

A role of lipoprotein(a) (lp(a)) as an ischemic stroke biomarker and as a predictor of recurrence has also been suggested [122]. Studies have shown an inverse relationship between high-density lipoprotein cholesterol (HDLc) and ischemic stroke, particularly in atherothrombotic subtype [123,124], wherein low HDLc concentrations are associated with increased stroke severity and poor clinical outcome [42]. It is of interest to consider the values of HDLc, along with those of LDLc, in order to better predict the risk of stroke. High-density lipoprotein (HDL) is well-known to play an antiatherogenic role in stroke [125], and this role may be partly promoted by counteracting the atherogenic effects of modified LDL, which was found to be increased in ischemic stroke, as discussed below. In this context, growing evidence has suggested that not only the concentration of lipids and lipoproteins but also the qualitative properties of lipoproteins that determine their role in stroke occurrence and atherosclerosis progression. The protective properties of HDL depend mainly on its physico-chemical properties, such as particle size. Elevated concentrations of small-sized HDL (HDL3), as well as of small dense LDL (sdLDL), were found to increase in ischemic stroke [44]. Meanwhile, a correlation was observed between HDL2 (large-sized) and carotid plaque thickness and area [126], and an inverse relationship was observed between small- and medium-sized HDL and stroke risk [127].

### 5.2. Lipoprotein Components

Besides size, the biological properties of HDL and LDL are highly influenced by their lipid and apolipoprotein (apo) composition and their content in hydrolytic enzymes. The plasma levels of these molecules have been suggested as putative biomarkers of ischemic stroke. Although their specific role in stroke subtypes is poorly defined, lipid-related biomarkers are suggested to be mainly involved in atherothrombotic stroke. Among apolipoproteins, the main candidates are: apoB/apoA-I ratio, apoE, and apoJ, also known as clusterin. A significant association between the increase in the carotid arterial intima-media thickness and increased apoB/apoA-I levels was found in patients with ischemic stroke [46,128]. Increased apoB levels are specifically associated with earlier onset of first-ever atherothrombotic stroke [129]; moreover, a high apoB/apoA-I ratio is described as a risk factor for this stroke subtype and it is increased in unstable plaques [130]. A positive dose-response association between apoE genotype and ischemic stroke was also observed [48]. Elevated plasma levels of apoJ are associated with decreased risk of stroke in younger healthy people [50], but its plasma concentration is increased in ischemic stroke, in animal models [131] and in human patients, and it is correlated with severity in the latter [132]. A proteomic analysis has found no changes in the amount of exchangeable apolipoproteins in the plasma lipoproteins from patients with carotid artery atherosclerosis [40]. In human carotid plaques, several apolipoproteins, are expressed including apo AI [47] and apoJ [51]. ApoJ and apoE are released by carotid plaques in a higher degree relative to their expression in non-atherosclerotic arteries [49].

Some enzymes associated with LDL and HDL have been proposed as putative ischemic stroke biomarkers, mainly, including platelet-activating factor acetylhydrolases (PAF-AH), also known as lipoprotein-associated phospholipase A2, and paraoxonase-1 (PON1). The circulating PAF-AH is bound to HDL and LDL, particularly in sdLDL [133]. PAF-AH, mainly when measured as mass, is considered as a risk factor for stroke occurrence and recurrence [52,53]. High serum PAF-AH mass levels have been described specifically in atherothrombotic stroke [54] and its increased concentrations correlate with plaque instability in both symptomatic and asymptomatic patients with carotid artery stenosis [134]. PAF-AH is highly expressed in macrophage-rich atherosclerotic plaques, wherein its expression is upregulated in advanced lesions, and in carotid plaque from symptomatic patients [55].

PON1 is a calcium-dependent HDL associated esterase that protects LDL against oxidation. Low levels of PON1 activity increase ischemic stroke risk [135], and they are positively associated with HDLc [136] and with unfavorable stroke outcome [137].

### 5.3. Modified LDL

As discussed, sdLDL levels are associated with ischemic stroke. This phenomenon could be a consequence of the higher susceptibility of sdLDL to suffer from modifications compared with normal-sized LDL. The inflammatory properties of modified LDLs are a main topic in atherosclerosis research. Although LDL can be minimally modified in plasma circulation, it becomes more easily modified when trapped in the arterial wall where it is exposed to enzymatic activities and free radicals released by cells. As a result of these modifications, LDL aggregates and thus its return into the circulation is hindered. Extracellular aggregated apoB-containing lipoproteins with atherogenic properties have been found in human carotid arteries [59]. The presence of aggregated LDL (aggLDL) in the circulation is considerably rare, but the concentration of this and other modifications, such as oxidized LDL (oxLDL), may increase in rupture-prone lesions. The plasma levels of oxLDL are particularly increased in large artery atherosclerosis and are related to poor functional outcome within 1 year after stroke onset [138]. Other studies have demonstrated the role of plasma oxLDL as a predictor of ischemic stroke outcome and recurrence, particularly in atherothrombotic subtype [56,57]. In addition, high levels of oxLDL in carotid plaque are correlated with unstable plaque [138,139] and are particularly increased in plaques from symptomatic patients [58].

Apart from oxLDL, the presence of a circulating form of modified LDL with inflammatory properties, called electronegative LDL (LDL(−)) has been described. LDL(−) is an LDL subfraction with a high negative charge, and it constitutes about 3−5% of the total LDL in healthy subjects. An increased proportion of LDL(−) was found in pathologies associated with cardiovascular risk, in patients with acute myocardial infarction [140], and even in the presence of subclinical atherosclerosis wherein there is correlation with the extent of carotid stenosis [141]. LDL(−) is a pool of LDL particles modified by several mechanisms that endow physico-chemical characteristics that differ from those of native LDL, such as increased aggregation level, increased apoJ, ceramide, and non-esterified fatty acid content, and increased PAF-AH activity [142]. In arterial wall cells, LDL(−) induces the release of several inflammatory mediators [142,143] that are putative biomarkers for ischemic stroke, as discussed below. It has been suggested that LDL(−) may serve as a marker of plaque vulnerability in ischemic stroke patients, who presented an increased proportion of this particle [144].

## 6. Inflammatory Biomarkers

Inflammatory factors have been studied extensively within the context of stroke. However, their use as biomarkers is hindered by the fact that these immunological factors are pleiotropic and that their levels may be elevated non-specifically in other conditions that mimic stroke, or in other diseases associated with an inflammatory response. In addition, some inflammatory biomarkers are associated with stroke per se and are directly related to infarct volume. However, stroke subtype may partly determine the inflammatory profile [145]. These putative biomarkers are especially important in the atherothrombotic subtype, in which inflammatory mediators within the plaque can be released into the circulation. As a result, cytokines, metalloproteinases, adhesion molecules, and cell receptors, among other factors, may be increased in the circulation and may be targeted for diagnosis and assessment of prognosis of atherothrombotic stroke, as described [13].

### 6.1. Cytokines

Several acute-phase proteins and inflammatory cytokines are increased in blood from patients with ischemic stroke, particularly the atherothrombotic subtype. High-sensitivity C-reactive protein (hs-CRP) levels are particularly high in symptomatic atherosclerosis [60], and they are associated with risk of ischemic stroke [146], poor outcome [147], and future recurrence of stroke [148]. Serum hs-CRP and interleukin (IL)-6 levels are correlated with echolucent unstable plaques [61]. Moreover, pentraxin 3 (PTX3) is described as an independent risk factor for the occurrence and severity of carotid atherosclerosis [62], and its increased levels in blood and plaque are correlated with carotid plaque instability [63].

Elevated levels of other cytokines, such as monocyte chemoattractant protein 1 (MCP-1), IL-2, IL-6, IL-9, IL-12, IL-18, and chemokine (C-X-C motif) ligand 1, are associated with ischemic stroke [64,70,71,149,150]. Some of them, such as MCP-1 and IL-6, are particularly increased in the atherothrombotic subtype, and they are correlated with severity, poor outcome, and recurrence [64,70,73]. Studies have reported a positive association between tumor necrosis factor alpha (TNF-α) and IL-1β with ischemic stroke risk [72] or future stroke recurrence [65]. In vulnerable plaques, IL-1β levels are increased in both plasma and carotid plaques, in which the expression levels of components of the inflammasome signaling pathway, involved in IL−1 β processing, are also increased [67]. IL-18 has not yet been found to be associated with stroke recurrence [65], but increased pre-stroke IL-18 levels may be related to the atherothrombotic stroke subtype [68]. IL-10, an anti-inflammatory cytokine, is usually decreased in ischemic stroke patients and is inversely associated with stroke risk and with poor outcome [149,151]. IL-23 is also highly expressed in carotid plaque and in serum from symptomatic patients and is associated with patient outcome [69]. IL-8 may also play a role in atherothrombotic stroke; however, contradictory findings have been obtained [64,152].

### 6.2. Enzymes, Adhesion Molecules and Cell Receptors

MMP are endopeptidases that degrade the extracellular matrix and thus they play an important function in inflammation, remodeling, and angiogenesis. Plasma MMP-2 levels have been found to be associated with stroke outcome [74]; however, MMP-2 concentration is inversely associated with the atherothrombotic subtype of stroke [73]. The main MMP related to the atherosclerotic process and ischemic stroke is MMP-9. High MMP-9 levels are associated with increased risk of mortality and major disability in ischemic stroke patients [71,77]. Moreover, MMP-9 levels are particularly increased in acute atherothrombotic stroke [78] and are higher in asymptomatic patients than in healthy subjects [39]. MMP-9 expression within carotid plaques is higher in vulnerable/symptomatic plaques than in stable ones [79,80]. Asymptomatic patients with vulnerable plaques also display high levels of serum neutrophil gelatinase-associated lipocalin (NGAL), which modulates the activity of MMP-9, and MMP-9/NGAL complexes [81]. High levels of MMP-7 have also been found in plasma and plaque from ischemic stroke patients, and they are related to mortality [75]. Increased concentrations of MMP-8, tissue inhibitor 1 of metalloproteinases (TIMP-1), and myeloperoxidase (MPO), an activator of MMP-8, have been observed in acute ischemic stroke. MMP-8 and MPO levels have been associated with stroke severity in the atherothrombotic subtype [76]. Moreover, MMP-8 is highly expressed in unstable plaques [153]. The plasma concentration of MPO was recently associated with ischemic stroke severity in non-lacunar stroke subtypes [82].

Cell adhesion molecules (CAMs) are located on cell surfaces and are involved in binding with other cells or with the extracellular matrix. In the inflammatory state, due to tissue damage, CAMs can be released and can activate the secretion of cytokines. The soluble (s) forms of selectins, including E-selectin and P-selectin, are elevated in ischemic stroke [154]. In atherothrombotic ischemic stroke, sP-selectin levels have been found to be elevated in the acute phase [84], and they are significantly correlated with sE-selectin levels in the subacute phase [83]. Studies have shown that apart from selectins, the soluble intercellular adhesion molecule 1 (sICAM-1) and soluble vascular adhesion molecule 1 (sVCAM-1) concentrations are increased in stroke patients [155].

Some cell receptors related to cell inflammation are increased in stroke patients. Lectin-like oxidized LDL receptor-1 (LOX-1) is a scavenger receptor found in atherosclerotic carotid lesions, the shedding of which is promoted by inflammatory molecules and by oxLDL [85]. The soluble form of LOX-1 (sLOX-1) can be released into the systemic circulation in ruptured atherosclerotic lesions [156] and may serve as an early diagnostic biomarker as in acute coronary syndrome [157]. Several studies, most of which have focused on carotid artery atherosclerosis, have found that sLOX-1 is increased in ischemic stroke, as reviewed by Hoffmann et al. [85]. Some studies have reported that the serum levels of sLOX-1 are particularly increased in the atherothrombotic subtype [86] and that they can predict patient outcome [87]. Skarpengland et al. [88] have reported that, in patients with carotid atherosclerosis, LOX-1 expression within the carotid plaques was increased relative to that in non-atherosclerotic vessels. Moreover, a study has reported that elevated sLOX-1 levels are associated with an increased stroke risk in asymptomatic patients [66].

Besides sLOX-1, the soluble forms of other scavenger receptors have been proposed as biomarkers for ischemic stroke. The elevated soluble cluster of differentiation (CD) 163 (sCD163) in plasma has been reported to be the result of macrophage activity within atherosclerotic plaques [92]. In ischemic stroke, sCD163 expression is increased, contributing to a worse patient outcome [91]. In carotid plaque, the expression levels of CD163 and CD36 are higher in symptomatic than in asymptomatic patients [39]. Other studies have corroborated the increased expression of CD36, a receptor involved in inflammation and foam cell formation, in carotid plaque, particularly in symptomatic and vulnerable carotid plaques [89,90], and in plasma from atherothrombotic stroke patients [90]. Increased sCD14 levels are associated with increased risk of stroke, probably in relation to its role in innate immunity and inflammation [158]. CD63 is a platelet receptor, the expression of which is increased in atherothrombotic stroke subtype [159].

### 6.3. Adipokines

There is evidence demonstrating the role of inflammatory adipokines in ischemic stroke, although it remains controversial [160]. Adiponectin levels have been found to be inversely associated with inflammatory markers [161]. All stroke subtypes show increased leptin levels and decreased adiponectin levels [162,163]. Resistin is positively correlated with stroke severity [164], ischemic stroke risk, and poor atherothrombotic stroke prognosis [97]. By contrast, low levels of anti-inflammatory omentin-1 have been found in ischemic stroke [95] and are related to poor functional outcome [96]. In the atherothrombotic subtype, patients with unstable carotid plaques showed significantly lower levels of serum omentin-1 than patients with stable carotid plaques. In this line, higher omentin-1 levels are inversely associated with carotid plaque instability, but they are not associated with moderate-severe carotid stenosis or occlusion [165]. Additionally, decreased levels of vaspin and ghrelin and increased levels of visfatin were observed in ischemic stroke patients [93,94], particularly in patients with high-grade carotid atherosclerosis [94]. The induction of visfatin in plaques was higher in symptomatic than in asymptomatic patients [166]. A study has shown that low apelin plasma levels are associated with atherothrombotic subtype [167]. Fatty acid-binding protein 4 (FABP4), an inflammatory adipokine that modulates lipid-signaling cascades, is increased in plasma and carotid plaques from symptomatic patients [39,98], and its expression is correlated with plaque instability [99].

## 7. Other Stroke Biomarkers

This review focuses on biomarkers that are related to inflammation and lipids. It is important to mention that not only their concentration but also genetic mutations and epigenetic mechanisms can strongly influence the predictive value of these biomarkers. Therefore, genetic analysis may add complementary relevant information to analyze protein levels or may be informative by itself. Single nucleotide polymorphisms have been found in several genes related to inflammation and ischemic stroke [168]; these polymorphisms include the following: IL-1β [169], IL-6 [170], IL-10 [171], MMP-2 [172], MMP-9 [173], MMP-12 [174], E-selectin [175], L-selectin [176], CD36 [177], PCSK9 [178], and adiponectin [179]. Related to lipoprotein components, some polymorphisms of apoB, PAF-AH, and PON1 are also associated with high ischemic stroke risk [180,181,182].

Apart from the previously mentioned biomarkers, the involvement of other biomarkers, such as those related to angiogenesis, thrombosis, and calcification has already been extensively reviewed elsewhere, in the context of carotid atherosclerosis [13]. Other putative stroke biomarkers related to ischemic stroke are brain damage proteins such as S100 calcium-binding protein B (s100b), neuron-specific enolase (NSE), light chain of neurofilament (NFL), tau, glial fibrillary acidic protein (GFAP), ubiquitin carboxy-terminal hydrolase L1 (UCHL-1), myelin basic protein (MBP), and brain-derived neurotrophic factor (BDNF) [183]. These biomarkers can only be found in the blood when there is severe brain damage, and they are not specific for atherothrombotic stroke and thus are beyond the scope of the current review. However, it may be interesting to take them into account when several molecules are analyzed together to generate a predictive score.

Other biomarkers that are gaining interest in their potential role in the diagnosis and monitoring of several diseases are microRNAs (miRNAs). However, at the moment, lack of research in large clinical trials may delay its implementation in the clinical setting. miRNAs are short, single-stranded, non-protein-coding RNA sequences that function as key post-transcriptional regulators. miRNAs are stable and detectable in peripheral blood, and they play a role in atherosclerotic plaque formation and stability [184] and in stroke [102]. Therefore, several miRNAs are described to be differentially found in the plasma from symptomatic and asymptomatic atherothrombotic stroke patients. In some cases, miRNA levels are dependent on the carotid plaque morphology, but not on stenosis degree [185]. The rest of the section focuses on the miRNAs upregulated or downregulated in plasma and/or in plaque in carotid atherosclerosis.

The miRNAs described herein have been found to be upregulated. miR-21 is one of the most validated miRNAs in carotid atherosclerosis. It is up-regulated in the serum from patients with ischemic stroke and asymptomatic atherosclerosis [100] and in human atherosclerotic plaques [101]. miR-130a-3p up-regulation was found to be related to carotid plaque progression in asymptomatic patients [104]. miR-133 was detected in the blood of patients with ICA. The expression levels of miR-133 and miR145 are higher in symptomatic carotid plaques than in the asymptomatic ones [105,106]. Other miRNAs up-regulated in carotid plaques and in blood from atherothrombotic patients are miR-143 [107], miR-155 [106], miR-199b [104] and miR-200c [109]. miR-494 is also up-regulated in the blood of stroke patients [102] and is abundantly expressed in unstable carotid plaques from CEA [112]. miR-221-3p concentration is lower in the symptomatic than in the asymptomatic cohort [110], but it is up-regulated in symptomatic carotid plaques [106] and in asymptomatic patients with stenosis progression [104]. miR-330-5p is up-regulated in unstable carotid plaque [186], but it is down-regulated in the serum from patients in the acute phase of stroke relative to that from healthy controls [103]. Other miRNAs are down-regulated in atherothrombotic stroke; these miRNAs include: miR-126 [107], the levels of which correlate with the degrees of cerebral atherosclerosis [187], miR-181b [108], miR-210 [101] miR-320b [111], and miR-638 [188].

## 8. Novel Therapies

Besides their diagnosis and prognosis use, blood biomarkers help in gaining a better understanding of the molecular mechanisms underlying ischemic stroke; also, they allow for the determination of putative targets for novel therapeutic interventions. In atherothrombotic stroke, targeting the initial specific factors that trigger the atherosclerotic process and an excessive inflammatory response is a promising therapeutic option. As an example, the toll-like receptor (TLR) pathway is essential in the activation of the innate immunity system, and it was found to be the most important activated pathway in the peripheral blood of stroke patients [37]. An increase in TLR4 activity following ischemic stroke was found to be associated with worse clinical outcome [189].

In the context of novel therapies, immunotherapy-based approaches that aim to improve stroke outcome are being developed. They are designed based on the inhibition of the acute innate immune response (to limit excessive tissue damage) and post-acutely stimulation of the adaptive immune system to limit post-stroke complications. A first line of therapy would be the use of anti-inflammatory drugs. In this regard, there are promising studies regarding the beneficial role of specific anti-inflammatory molecules, such as IL-10, in atherosclerosis, but they need to be further studied and are still not clinically used in the treatment of patients. Regarding the inhibition of acute innate immune response, in some preliminary studies, the action of inflammatory cytokines is blocked by using different approaches: the use of soluble receptors, the inhibition of their synthesis, the use of anti-inflammatory molecules, or the use of monoclonal antibodies against specific inflammatory mediators. In most cases, however, those studies are preliminary, and the treatments yielded modest beneficial effects due to the complexity of the immune response; conducting a risk-benefit analysis is challenging, and the clinical testing of these treatments has only just started. In this context, clinical trials involving the use of antibodies to block the interaction of leukocytes with endothelial cells have demonstrated limited clinical translation [190]. The CANTOS trial, which has focused on the effect of blocking IL-1β by the monoclonal antibody canakinumab, has demonstrated the beneficial effect of this approach in myocardial infarction patients, regardless of its lipid-lowering effect [191]. However, its effect is limited in stroke and further studies are warranted. In mice, the post-ischemic administration of canakinumab has improved the outcome of stroke [192]. In humans, a preliminary study has shown that a receptor antagonist of IL-1β (IL-1RA) has improved stroke outcome [193]. Studies of blocking of TNF-α, demonstrated a neuroprotective result in animal models and humans, as reviewed by Tuttolomondo [194]. ADAM17, an inhibitor of MMP, is also considered as a potential stroke therapy because it diminishes TNFα production [195]. In addition, a first line of therapy would be the use of anti-inflammatory drugs. In this regard, promising studies show the beneficial role of anti-inflammatory molecules, such as IL-10 or colchicine, in atherosclerosis, but they need to be further studied and they are still not used in the treatment of patients. Taken together, there are no clear immunotherapies that offer protection from damage after acute ischemic stroke; in this line, a deeper knowledge of the immune response to ischemic stroke is needed in order to better evaluate the effects of these therapies from which patients may benefit.

It has been hypothesized that some chronic bacterial infections may be associated with the development of atherosclerosis and the risk of complications, such as myocardial infarction or stroke. These bacterial infections seem to play a role in the initiation, progression, and the destabilization of atherosclerotic plaques. In this regard, sub-antimicrobial dosages of antibiotics could be beneficial in inhibiting inflammation. This is suggested by some studies demonstrating the benefits of antibiotics in selected patients. A study from Sander et al. showed a protective effect of roxithromycin treatment in the progression of arteriosclerosis [196]. Once again, a better knowledge of plasmatic biomarkers associated with chronic-infection-immune response will probably help in selecting patients who are candidates to receive antibiotic drugs.

Regarding the pivotal role of lipids in atherothrombotic stroke, LDL and modified LDL are potentially important targets. A recent study has reported that PCSK9 administration in combination with statins has efficiently lowered LDLc levels and has reduced the operative complications of carotid stenting probably by stabilizing carotid artery plaque [197]. Other future therapies aiming to reduce the modification of LDL and/or to improve the qualitative properties of LDL and HDL may be very considerably valuable in preventing carotid atherosclerosis progression and complications.

## 9. Plasma Biomarkers in the Clinical Practice and Future Directions

Currently, there is no evidence for using a specific plasma biomarker as a predictor of carotid plaque vulnerability or as a predictor of stroke recurrence in patients with carotid atherosclerosis. However, the CANTOS trial represented a proof-of-concept that a plasmatic inflammatory biomarker (in that case CRP) can be used for selecting patients with atherosclerosis who may be candidates for immunomodulation therapies. Despite the little specificity of the CRP, elevated levels of this inflammatory biomarker identified patients that benefited from treatment with canakinumab. These results encourage the research on plasmatic biomarkers. We think that future translational research comprising plasmatic biomarkers and carotid atherosclerosis should be focused on three main frameworks:

(1) Testing the association between circulating biomarkers and clinical outcomes in big cohorts. Although little observational studies are useful to drive hypothesis, they have to be tested ultimately in big cohorts, in order to increase the sample size. In this regard, we would encourage basic and translational researchers to partnerships with clinicians in big clinical cohorts or trials. Biomarkers substudies within big cohort studies or trials are warranted to increase the evidence of the predictive value of circulating biomarkers.
(2) Studying the association between circulating and imaging biomarkers. There are many validated imaging biomarkers that are currently used to predict carotid plaque vulnerability. Studying the association between these validated imaging biomarkers and circulating molecules can be a useful intermediate step for selecting good candidates before testing their predictive value in big cohorts.
(3) Including circulating biomarkers in predictive scores. In the field of stroke, there are several examples that demonstrate that merging information from clinical, imaging, and laboratory variables increases the predictive power of some scales created to predict stroke recurrences. We believe that this approach should be taken into account in future research as it may increase the chances of a hypothetical implementation of circulating biomarkers in the clinical practice.

## 10. Conclusions

In summary, circulating biomarkers profiling is a promising tool that may help in the future in identifying plaque vulnerability in patients who are at high risk of recurrence or even first-ever atherothrombotic stroke and would benefit from carotid revascularization. Unfortunately, in spite of the extensive up-to-now search for circulating biomarkers in stroke, no single reliable individual biomarker has demonstrated enough sensitivity and specificity to be established in the clinical practice yet. The most feasible possibility would be to find a combination of several molecules in order to develop a panel of biomarkers. In atherothrombotic stroke, these biomarkers are expected to be lipid-related and inflammatory molecules, which are distinctive features of this etiology, and they may be released from the carotid plaque into the circulation as a consequence of plaque instability or rupture. In this scenario, there is growing evidence of the role of inflammatory biomarkers, as they arise as a consequence of acute stroke, but without being predictive of the progression of the disease. Moreover, their concentration may be non-specifically elevated in other concomitant pathologies. In the context of atherothrombotic stroke, biomarkers related to lipoproteins may be more specific and predictive than inflammatory molecules. In this regard, it would be important not only to measure the concentration of lipids and lipoproteins but also to study the role of alterations in the qualitative properties of lipoproteins, particularly those leading to the presence of modified forms of LDL. However, a main drawback is that the assessment of some of these properties is still technically difficult to implement in routine laboratories. Prospective large clinical studies applying a combination of circulating and imaging biomarkers together with clinical data are essential. Taken together, there is still a long way in the search for circulating biomarkers easily measurable in the clinical practice that may be useful to select patients at high risk of atherothrombotic stroke. In a broader perspective, some of these biomarkers might also be useful in other cardiovascular diseases associated with atherosclerosis.

## Figures and Tables

**Table 1:** Main candidate biomarkers found in plasma/serum and/or plaque from atherothrombotic stroke patients.

		Basal State/Asymptomatic	≤24 h	≤14 d	≥14 d	Plaque	References
Lipid-related and lipoproteins biomarkers	TC		P	P			[41,42]
	LDLc		P	P	P		[41,42,43]
	HDLc		P	P	P		[41,42]
	HDL3	P					[44]
	PCSK9	P				Yes	[45]
	apoA-I		P, S	P, S		Yes	[46,47]
	apoE		P	S	S	Yes	[48,49]
	apoJ	P		S	S	Yes	[49,50,51]
	PAF-AH	S	S	S		Yes	[52,53,54,55]
	oxLDL		P	P		Yes	[56,57,58]
	aggLDL					Yes	[59]
Inflammatory biomarkers	hs-CRP	S	P, S	P, S			[60,61]
	PTX3	S	P	P	S	Yes	[62,63]
	IL-6	P, S	P, S	P, S		Yes	[64,65,66]
	IL-18	S	S			Yes	[67,68]
	IL-23				S	Yes	[69]
	MCP1	P, S					[70]
	TNF-α	P	P *	P *		Yes	[64,65,66,71,72]
	MMP-2		P	S		Yes	[66,73,74]
	MMP-7				P	Yes	[75]
	MMP-8		S				[76]
	MMP-9		P, S			Yes	[71,77,78,79,80]
	NGAL	S					[81]
	MPO		P, S	P			[76,82]
	E-selectin			P *	P *	Yes	[83]
	P-selectin		P *	P *	P *		[83,84]
	LOX-1	P	S	P	P	Yes	[66,85,86,87,88]
	CD36				P	Yes	[39,89,90]
	CD63		P				[91]
	CD163		S			Yes	[39,92]
	Ghrelin		P, S				[93,94]
	Omentin-1		S				[95,96]
	Resistin	P	P				[97]
	Vaspin		P, S				[93,94]
	FABP4				P	Yes	[98,99]
miRNA	miR-21	S		S		Yes	[100,101]
	miR-126		P	P	P		[102,103]
	miR-130a	P	P	P			[102,104]
	miR-133		S			Yes	[105,106]
	miR-143/145		P	P		Yes	[102,105,106,107]
	miR-155	P				Yes	[106]
	miR-181b		P	P			[108]
	miR-199b	P					[104]
	miR-200c	P				Yes	[109]
	miR-210	P	P			Yes	[101]
	miR-221	S				Yes	[106,110]
	miR-320b	S	P				[102,111]
	miR-330	S				Yes	[103,110]
	miR-242		P			Yes	[102]
	miR-494		P				[102,112]

P: Plasma; S: Serum; * Contradictory information.
